# Aphid Assemblages Associated with Urban Park Plant Communities

**DOI:** 10.3390/insects12020173

**Published:** 2021-02-17

**Authors:** Tadeusz Barczak, Janina Bennewicz, Maciej Korczyński, Małgorzata Błażejewicz-Zawadzińska, Hanna Piekarska-Boniecka

**Affiliations:** 1 Department of Biology and Animal Environment, UTP University of Science and Technology in Bydgoszcz, Hetmańska 33, 85-039 Bydgoszcz, Poland; janina.katarzyna.szajda@gmail.com (J.B.); malba@utp.edu.pl (M.B.-Z.); 2 Department, UTP University of Science and Technology in Bydgoszcz, Polish Botanical Society, Al. Prof. S. Kaliskiego 7, 85-796 Bydgoszcz, Poland; malerudy@wp.pl; 3 University of Life Sciences in Poznań, Department of Entomology and Environmental Protection, Dąbrowskiego 159, 60-594 Poznań, Poland; hanna.boniecka@up.poznan.pl

**Keywords:** plant communities, aphid assemblages, Aphididae, habitat effect, urban green environments

## Abstract

**Simple Summary:**

Some aphid species are important as pests on plants growing in urban areas. The aim of the study was to determine and compare plant communities and the assemblages of aphids associated with them in different urban park habitats. The study was carried out in Bydgoszcz (northern Poland), and four parks were taken into consideration. We have indicated plant communities and plant species most affected by aphids and the least. for example pear, elderberry, maple and jasmine were among those most affected by aphids, and acacia was not. This should be taken into account for aesthetic reasons when arranging parks. The density of buildings turned out to be the factor that differentiated the most from aphids.

**Abstract:**

Some aphid species are important agricultural pests, sometimes also found on plants growing in urban areas. In this work, we set out to identify the plant species, communities or habitats that are more attractive to aphids in order to limit their spread into new green areas. The aim of the study was to determine and compare plant communities and the assemblages of aphids associated with them in different urban park habitats. The research hypothesis assumed that the differences between aphid assemblages depend on plant diversity and hence reflect urban park habitat environmental conditions, in particular the plant communities and the floral structure. The study was carried out in Bydgoszcz (northern Poland), and four parks were taken into consideration. Herein, floristic lists were used to calculate ecological indicator values for each park. The aphid species richness was determined, as well as the relative abundance and dominance structure similarities of the aphid assemblages. Our results demonstrated that *Prunus* spp. were strongly infested by *Hyalopterus pruni*, similarly as *Philadelphus inodorus* by *Aphis fabae*, *Sambucus nigra* by *Aphis sambuci*, and *Acer* *platanoides* and *A. pseudoplatanus* by *Periphyllus testudinaceus*. Park plantations of *Robinia pseudoacacia* were not very attractive to aphids. The most attractive plant communities to aphids were syntaxonomically identifiable as alluvial alder forests in the layer of trees and *Cornus sanguinea* in the layer of shrubs.

## 1. Introduction

Urbanisation can promote biodiversity by increasing habitat diversity and providing new refugia and movement corridors for wildlife [1,2,3,4,5]. In contrast, Jones and Leather [6] have demonstrated the negative impacts of roadsides on invertebrate populations in the cities. Urban green spaces are therefore vital for the local ecology, and consequently, it is reasonable that the ecological value of these areasshould be included in urban planning and investments [6,7,8] so as to improve the degree of environmental sustainability [9]. McIntyre et al. [10] noted that invertebrates are important in the cycling of organic matter, nutrient transport, soil aeration and pollination. Thus, they influence the ecosystem function of urban areas. Furthermore, invertebrates such as aphids are a food source for higher trophic levels (predators, parasitoids and birds), who will even use honeydew as a nutrient. Thus, changes in aphid numbers can influence both these organisms as well as plants [11,12,13,14].

Aphids are common insects, and some species are important pests of many crops and are also found on plants growing in urban areas, including alien species to Polish flora [15]. The number of aphid species and their abundance may vary depending on, for example, the habitat conditions and plant diversity. The abundance of aphids varies significantly because of the type of land management practised and the levels of anthropogenic pressure. Furthermore, the urban ecosystem effects populations of aphids as well [16]. The specific urban climate, prolonged periods of elevated temperatures, changes the phenology of host plants and the biology of insects. Regarding aphids, this is beneficial as it results in an increase in the number of generations and hence the size of their colonies. Insects with piercing and sucking mouthparts, such as aphids, are one of the most numerous groups of city-dwelling arthropods and, at the same time, the most threatening to urban flora [17,18]. Aphids, visible on leaves, and giving off honeydew, stain plant leaves and reduce the aesthetic values of urban greenery [19]. Sucking insects are especially abundant on trees growing along the streets, even hundreds of times higher compared to trees in parks or in suburban areas [20,21,22]. The most important factors responsible for changes in the phenology of aphids include temperature, shortage of water and quality of food—the host plant species [23,24,25,26]. It should be noted that aphids reproduce only within a certain temperature range [27].

Most of the work on aphids in urbanised environments in Poland concerns listing the species inhabiting particular host plants [28], including those in city green areas [5,20,29,30]. In contrast, the associations between aphids and plant communities are analysed less often [21].

The fundamental aim of the study was to determine and compare plant communities and the assemblages of aphids associated with them in different urban park habitats. An additional aim was to indicate those plant species, communities or habitats that are more attractive to aphids in order to propose to limit their plantings in newly arranged green areas.

The research hypothesis assumed that the differences between the aphid assemblages depend on the plant diversity, which, in turn, is a reflection of the environmental conditions of urban park habitats, in particular their flora structure and plant community associations.

## 2. Materials and Methods

### 2.1. Study Area

**Figure 1:**
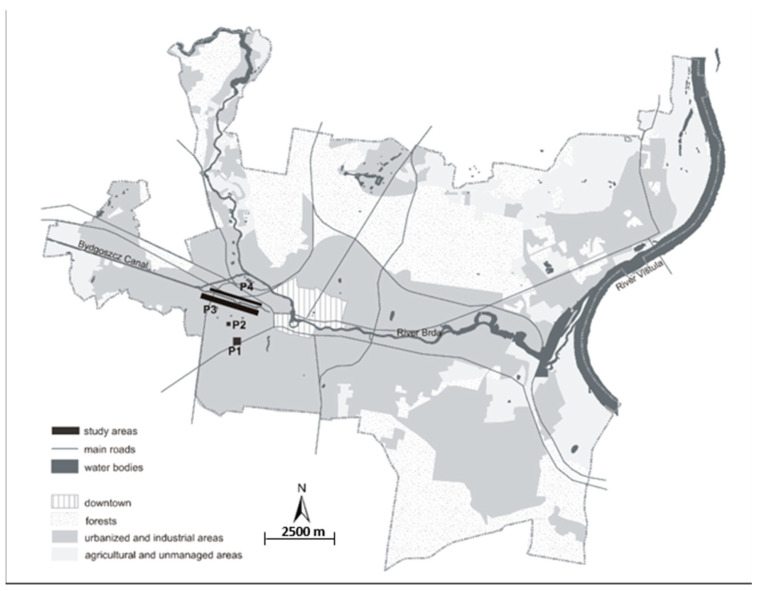
A map of Bydgoszcz, Poland (a scheme). Legends: Błonie residential district (P1), Park Księżycowy (P2), Park Nad Starym Kanałem on dry soil (P3) and Park Nad Starym Kanałem on moist soil (P4).

Our research was carried out in four city parks in Bydgoszcz, northern Poland. They are located on the north–south axis running from residential areas on the plateau towards the parks near the city centre in the Brda River valley (Figure 1).

The southernmost park, of 2.4 ha surface area, named Błonie Residential District (P1), is surrounded by residential buildings, mainly four-storey apartment blocks. The park includes mown lawns with single trees, and shrub beds near buildings.

Another study area, of 1.5 ha area, Park Księżycowy (P2), is located northwards, also in a residential district. It was established on a slope of an upland terrestrial feature, with a relative elevation of 35 m, oriented towards the northern direction. The central point of the park is a pond formed on the site of a small spring. The park is dominated by lawns and single trees and is surrounded by single-family houses.

The next park, named Park Nad Starym Kanałem, has a clearly different character. It stretches on both sides of a now unused arm of the Bydgoszcz Kanał. Park Nad Starym Kanałem is located in the western part of the city, 2 km from the centre of Bydgoszcz, separated from it by the Bydgoszcz Kanał. Trees dominate the park, and the lawns here are rarely or never mown. In physiographic terms, the park consists of two different parts. The first part, the marginal part of the park, is a relatively dry habitat, with a lower density of tree stands, while the ground cover in the park is formed by grassland communities. This part of the park was the third study site and is named Park Nad Starym Kanałem on dry soil (P3). P3 is a green belt on the southern embankment of the Bydgoszcz Kanał, covering a 3.60 ha green area with a length of 3 km. The second part of Park Nad Starym Kanałem directly adjoins the canal and includes small patches of grassland, dominated by macroforbs. It is established on sandy moist soil that had been transformed during the construction of the Bydgoszcz Kanał. This part of the park was the fourth study site and is called Park Nad Starym Kanałem on moist soil (P4). P4 is the narrow strip of green on the northern embankment of the Bydgoszcz Kanał, covering an area of 8.20 ha and extending over a length of 1.5 km and a width of 50–100 m (Figure 1).

### 2.2. Methods of Botanical Research

The vegetation in the studied parks was analysed once in the growing season of 2012. Our earlier observations indicated a small floristic variability within the studied parks and their surroundings. Complete floristic lists with coverage degrees (in %) according do Braun-Blanquet [31] for individual species were prepared for each park. Plants were pressed, dried and later identified to the species or genus level according to Rutkowski [32], but systematic names followed Mirek et al. [33]. We calculated the collective participation of the most important syntaxonomic classes and values of the Shannon–Wiener diversity index in each park using the *H*’ index, while assessing its value according to Shannon’s formula [34]. We then used these floristic lists to determine ecological indicator values for each park [35] and the degree of anthropogenic transformation of the flora using the anthropophytisation rate [36] (see Table 1). This is indicated by the percentage of alien species in the flora. Ellenberg‘s indices for each park express the weighted average of indices for individual floristic lists. The value of indicators for light (L), temperature (T), continentality (K), reaction (R) and nitrogen (N) may change from 1 to 9 and for moisture (F) from 1 to 12. They are directly proportional to the intensity of the ecological factor. The percentage share of alien species in the flora of parks determines their degree of anthropogenic transformation. Floristic lists were also used to classify the parks based on the calculated percentage similarity and unweighted pair group method with arithmetic mean (UPGMA). Ordination of parks was carried out using canonical correspondence analysis (CCA) case scores (Euclidean) and indicator values by Ellenberg et al. [35] as environmental parameters [37]. CCA and UPGMA were applied to quantify and test the association between parks through plant community composition variation and environmental properties. The MVSP statistical software package [36] was used for data analysis. The classification of plant communities followed the system of Matuszkiewicz [38].

We applied Pearson’s test [39] for linear correlation to assess the significance of differences between correlation coefficients calculated for ecological indicators, as proposed by Ellenberg et al. [35], and the positions of studied parks on the ordination axes of CCA [40,41,42].

### 2.3. Methods of Entomological Research

Studies on aphids were conducted in 2012-2014 in Bydgoszcz, northern Poland. We investigated aphids of the Aphididae family by observing their colonies on various aphid host plant species in a given search area and collecting specimens for the determination of species and aphid assemblages. Observations of the occurrence of aphids were conducted every 10 days by monitoring aphid host plants from the moment of their appearance (the end of April) to the beginning of August, when aphids usually disappear. All plant species that are potential aphid hosts were monitored, while only random samples/leaves were taken from plants infested by aphids. Each time, the aphids were counted in three replicates on different plant specimens of the same host plant species that were colonised by aphids in a given habitat. To count aphids, one sample was one leaf with one colony of a given aphid species on randomly chosen plant specimens, or in the case of *Pinus* spp., one sample was aphids on the final section of the stem with needles, and in the case of *Robinia pseudoacacia*, one so-called composite leaf with aphids. To estimate the number of aphids in the colony, we took into consideration 30 samples per aphid host plant species every year in a given type of habitat/park.

Additionally, in the parks, from each sample/colony, several aphids were placed separately in a plastic bag and transported to the laboratory to determine the aphid species. Live aphids were killed and preserved for identification in 75% ethanol and identified in the laboratory. Aphids were analysed down to species level using a system for classification of aphids by Blackman and Eastop [43], and Szelegiewicz [44] and Taylor [45], as the keys to identify the aphids. Where it was necessary, we classically made preparations with aphid morphs and determined them according to the keys. The aphid species richness was determined, as well as relative abundance (D, dominance) and the dominance structure similarities of the aphid assemblages between the compared studied areas/parks.

In accordance with Klimaszewski et al. [46], the following domination classes were used to identify the relative abundance of each aphid species: D4, very numerous species, dominant, accounting for more than 20% of the collected material in the given habitat; D3, numerous species, subdominant, representing 10.1–20% of the total number of specimens; D2, quite numerous species, recedent, ranging from 3–10% of the collected material; and D1, rare species, subrecedent, representing less than 3% of the total number of individuals. This classification has been made on a fixed number of plants of the same species, so a comparison occurred between an equal number of plants.

Quantitative comparison of the aphid assemblages was calculated using the Renkonen‘s coefficient of dominance similarity, Re. It was assumed that assemblages are similar for values of Re from 50% onwards [47].

Re = ΣD_min._,

where *D*_min._ is the minimum value of relative abundance for a given species in two compared aphid assemblages.

For a qualitative–quantitative comparison, the Hutcheson test [48] was used, where the Shannon–Wiener index (*H*’) [34] was applied for the assessment of the significance of differences between values of *H*’ of the compared aphid assemblages between the parks, taking into consideration a normal distribution of Student’s *t*-test.

CCA and UPGMA were applied to quantify and test the association between parks through aphid assemblage composition variation and environmental parameters and to estimate the potential co-existence of different aphid species in the aphid assemblage in a given study area/park [35,49,50]. CCA arranges the sites in geometric space producing a scatterplot in which points (i.e., habitat unit samples or taxa) are systematised so that the points close together correspond to sites with a similar species composition of plants and aphids and points that are far apart correspond to sites that are dissimilar in species composition [51].

## 3. Results and Discussion

### 3.1. Flora in the Parks

**Figure 2:**
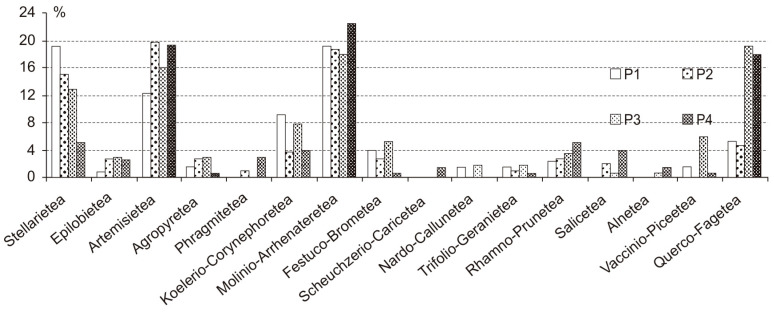
Main syntaxon characteristic groups share. Legends: Błonie residential district (P1), Park Księżycowy (P2), Park Nad Starym Kanałem on dry soil (P3) and Park Nad Starym Kanałem on moist soil (P4).

Flora in the studied parks was formed by 325 vascular plant species. The highest species richness was found for P3 (see abbreviations in the Material and Methods section above). The lowest number of species was reported for P2, which was also characterised by the lowest Shannon–Wiener diversity index (*H*’) despite similar calculated values (Table 1). The most important plant taxa were those typical for meadows from the *Molinio-Arrhenatheretea* class and ruderal species from the *Artemisietea* class (Figure 2). The flora of parks was characterised by the decreasing share of species typical of arable fields and habitats at the early stages of succession in phytocoenoses from the *Stellarietea* class. Aquatic plants in the syntaxonomical spectrum were represented by helophytic *Phragmitetea* communities in P2 and in the moist part of P4 along a section of the Bydgoszcz Kanał. The high density of trees in P4 and P3 created favourable conditions for the growth of plants associated with *Rhamno-Prunetea* thickets and forests. Species typical of *Vaccinio-Piceetea* pine forests and *Querco-Fagetea*, *Alnetea* and *Salicetea* deciduous forests were also recorded. There were clear differences between the investigated parks in terms of the index of anthropogenic transformation (Table 1). This index shows the percentages of the alien Scheme 1 and P2, both surrounded by residential buildings. The most common alien species there included *Acer negundo* Linnaeus, *Lycium barbatum* Linnaeus, *Malus domestica* Borkhausen, *Prunus cerasifera* Ehrhart, *Quercus rubra* Linnaeus, *Robinia pseudoacacia* Linnaeus and *Rosa rugosa* Thunberg [52,53].

**Figure 3:**
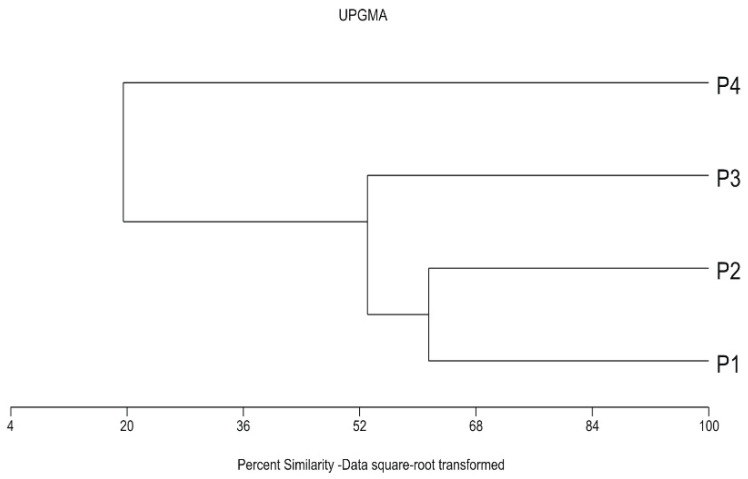
Dendrogram of similarity of flora in the parks (the analysis was performed with Euclidean distance, unweighted pair group method with arithmetic mean (UPGMA), as the measure of similarity). Legends: Błonie residential district (P1), Park Księżycowy (P2), Park Nad Starym Kanałem on dry soil (P3) and Park Nad Starym Kanałem on moist soil (P4).

Two parks (P1 and P2) were similar in syntaxonomic terms (Figure 2). The dominant plant community there was *Lolio-Polygonetum*, found on lawns. There was also a significant share of grasses commonly used in lawn seed mixtures (*Poa pratensis* Linnaeus, *Festuca rubra* Linnaeus). The sandy soil in P1 creates favourable conditions for the growth of plants associated with grasslands on poor soils, such as *Sedum acre* Linnaeus, *Trifolium arvense* Linnaeus and *Festuca ovina* Linnaeus. Park P3 is intermediate between P1 and P2, and P4 (Figure 2 and Figure 3). Wide lawns under the canopy of a high pine stand dominate. Coniferous forest species were found only here in P3. Moreover, plants typical of deciduous forest habitats colonise the clumps of trees and shrub-growths. This makes it floristically similar to park P4. The latter syntaxonomically refers to the alluvial alder forests of *Alnus glutinosa* (L.) Gaertner in the layer of trees and *Cornus sanguinea* Linnaeus in the layer of shrubs. The characteristic species Phragmitetea, Molinio-Arrhenatheretea, Salicetea, Alnetea and Querco-Fagetea are very important in the floristic structure.

The study site by the Bydgoszcz Kanał, defined as P4, is dominated by a lime-oak-hornbeam forest from the *Tilio-Carpinetum* class and, on some patches, the ash-elm floodplain forest *Ficario-Ulmetum*. The tree layer is formed by *Alnus glutinosa* (Linnaeus) Gaertner, *Acer platanoides* Linnaeus, *Tilia cordata* Miller and *Acer negundo* Linnaeus. Under the tree canopy, the highest rate of cover was found for *Sambucus nigra* Linnaeus, *Cornus sanguinea* Linnaeus, *Aegopodium podagraria* Linnaeus, *Alliaria petiolata* (Marschall von Bieberstein) Cavara & Grande and *Ficaria verna* Hudson. In the dry part of this park (P3), the herbaceous layer under the canopies of *Pinus sylvestris* Linnaeus is formed by *Festuca trachyphylla* (Hackel) Krajina, *Agrostis capillaris* Linnaeus and *Trifolium arvense* Linnaeus, and only a few species typical of coniferous forests. Patches of *Lolio-Polygonetum* are found on sites where lawns are managed more frequently. In contrast to the forest part on moist soil (P4), the dry part (P3) has features more characteristic of a typical park.

**Figure 4:**
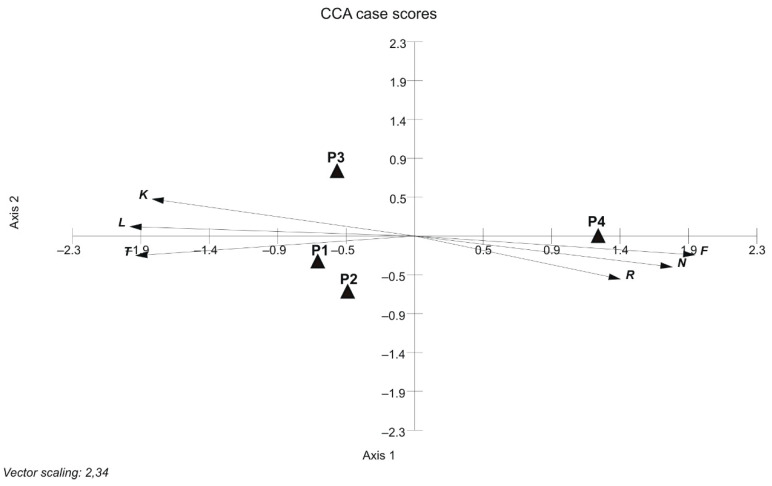
Ordination diagram based on canonical correspondence analysis (CCA) of the investigated parks’ flora (to reveal ecological gradients, Ellenberg et al. [35] indicator values were plotted: L—light; T—temperature; K—continentality; F—moisture; R—reaction; N—nitrogen). Legends: Błonie residential district (P1), Park Księżycowy (P2), Park Nad Starym Kanałem on dry soil (P3) and Park Nad Starym Kanałem on moist soil (P4); axis 1—vertical line, axis 2—horizontal line.

Separation of the vegetation of the parks is confirmed by the average values of Ellenberg’s environmental indicators [35]. The calculated climatic values (L, K, T) are the lowest for park P4 (Table 1). They indicate that it is the least sunny, the coldest and the most stable with regards to temperature. Edaphic indicators characterise it as the wettest, with higher soil pH and higher fertility. The CCA ordination clearly illustrates these features. The mentioned attributes are correlated with axis 1 (Figure 4). The location of park P3 on axis 2 confirms its indirect character between parks P1 and P2 and park P4.

The diversity of the investigated parks was confirmed by numerical analyses. Both the ordination and classification analyses pointed to park P4 as a separate unit (Figure 3 and Figure 4). Considering floristic qualitative features, the greatest similarity was found between parks P1 and P2 due to the physiognomic dominance of species commonly used in lawn seed mixtures. Dry and poor soils were habitats for xeromorphic and oligotrophic species present in the flora of parks P1 and P3. The ordination analysis using CCA distinguished the moist part of park P4 established directly along the Bydgoszcz Kanał. Its position along axis 1 was mainly due to habitat elements such as light (L), temperature (T) and moisture (F). There was a significant correlation between the calculated ecological indicator values [35] and the coordinates of individual parks on axis 1 of the CCA diagram (Table 2, column A; Figure 4). The calculated Pearson’s correlation coefficients, L = −0.992, T = −0.996 and F = 0.969, were greater than their critical values at *p* ≤ 0.05. Axis 2 of the diagram was not correlated with individual ecological indicators. However, the strongest correlation values, i.e., −0.45, 0.32 and 0.35, were obtained, respectively, for reaction R, nitrogen (N) and continentality (K) (Table 2, column A; Figure 4). Here we can observe the effect of environmental variables (long arrows) on the plant community structure. The direction of each arrow (environmental variable) points to an increase in that particular variable. The longer the arrow, the stronger effect that a particular variable had on the plant community structure. Plant species that are located near each other were influenced in a similar manner by the different environmental variables. For example, the ordination is divided into two groups of parks to assist in generally defining each one of them by the variables within it and to identify the parks [54].

### 3.2. Aphid Assemblages

The study conducted in 2012-2014 revealed the presence of 66 aphid species on 75 plant species out of over 300 found in the investigated parks. The infestation of plants by aphids was the strongest in P4 (Table 3 and Table 4), on moist soil with rich vegetation, under the canopies of deciduous trees, where 32 aphid species were found feeding on 38 plant species. A similar abundance of aphids (Table 3 and Table 4) was recorded in Park Księżycowy (P2), adjacent to home gardens. Here, 29 aphid species colonised 30 plant species. In the park on dry soil (P3), where pine was dominant and conditions for aphids were less favourable, the infestation of plants was about 2/3 lower than in P4, where 28 aphid species fed on 32 plant species (Table 4). P1, surrounded with closely set residential blocks, despite its diverse flora formed mainly by trees and shrubs, does not appear to be an attractive foraging site for aphids. However, here we should add that it is also the park with the highest number of exotic plants, so the finding is rather normal, considering that aphids are insects typical of temperate-cold areas [43].

The three-year study showed the lowest number of plant species (28) infested by the lowest number of aphid species (25) and the smallest colonies in P1, which confirms the hypothesis that aphids in such a densely developed residential district have less favourable living conditions (Table 3 and Table 4; see also [23,24,25,26,55]). 

During the whole study period, *Hyalopterus pruni* Geoffroy was the most abundant aphid species in parks P2 and P4. This aphid was found on *Prunus domestica* Linnaeus in all the investigated parks, on *Prunus cerasifera* Ehrhart in all parks but P2 and on *Phragmites australis* (Cavanilles) Trinius & Steudel, the secondary host of *H. pruni*, in P2 and P4 (Table 3). Another abundant species was *Aphis sambuci* Linnaeus feeding on *S. nigra* in P2, P3 and P4. *Periphyllus testudinaceus* Fernie was recorded on various *Acer* trees in all the investigated parks, while *Aphis fabae* Scopoli-complex was found on *Cirsum arvense* (Linnaeus) Scopoli and *Philadelphus inodorus* Linnaeus in P1, on *Arctium* spp., *P. inodorus*, *Rumex acetosa* Linnaeus and *Viburnum opulus* Linnaeus in P2, on *Angelica archangelica* Linnaeus subsp. *litoralis* (Fries) Thellung, *Arctium minus* (Hill) Bernhold, *Cirsum arvense* (Linnaeus) Scopoli and *Euonymus europaea* Linnaeus in P4 and only on *Rumex crispus* Linnaeus in P3; *Myzus cerasi* was recorded on *Cerasus* spp. and *Galium aparinae* only in P2 and P3 (Table 3).

*R. pseudoacacia* is a species native to the south-eastern United States and is not very attractive to aphids in Poland. Hence, it would seem to be more suitable for planting in parks than other trees species. It should also be added that this species is not among the most numerous non-native plant species in Poland [52]. However, *R. pseudoacacia* is an alien for the flora of Europe, and Poland especially, and its cultivation and dissemination in horticulture is banned in the European Union [52].

In every park, such aphid species as *H. pruni* Geoffroy *on P. domestica, P. cerasifera, A. fabae* on *P. inodorus*, *A. sambuci* on *S. nigra* and *P. testudinaceus* on *A. platanoides* and *Acer pseudoplatanus* Linnaeus formed numerous colonies on them, profusely secreting honeydew and causing the leaves to be dirty and deformed, which is often compounded by the visiting colony of ants [12,14,56]. Urban environments are a valuable source of food for many groups of aphidophages, including predators, as well as parasitoids, first, because of the presence of aphids colonies and, second, because nectar or honeydew is a source of carbohydrates [12,14,57,58,59,60]. Aside from exploiting nectar or honeydew as an adult food source, aphid natural enemies, including hyperparasitoids, can also use honeydew as a host location kairomone and an oviposition stimulus [12,61].

Three dominant aphid species were identified: *H. pruni*, *Macrosiphum rosae* Linnaeus and *P. testudinaceus* in P1; *A. sambuci*, *H. pruni* and *Myzus cerasi* Fabricius in P2; *Eucalipterus tilliae* Linnaeus, *M. cerasi* and *P. testudinaceus* in P3; and two species, *H. pruni* and *P. testudinaceus,* in P4. Other aphid species were abundant in individual years, but their total participation over the 3-year study did not exceed 10% of the entire assemblage (Table 5). 

The quantitative comparison of the aphid assemblages in the studied parks made for 2012–2014 based on the Renkonen index (Re) showed a similarity between the assemblages in P2 and P4 in terms of the dominance structure. In 2012, the Re value was greater than 50% when comparing P4 and other parks (P1, P2 and P3), which indicates a similar dominance structure of aphid assemblages associated with these parks. In subsequent years, there was no quantitative similarity in the assemblages of aphids between the studied green areas in Bydgoszcz (Table 4).

In 2012–2014, aphid assemblages in P1 and P3 had the greatest species diversity (*H*’ = 3.9) compared to the assemblages in P2 and P4 (Table 4). However, there were no significant differences in the species diversity index (*H*’) between aphid assemblages assessed using the Hutchison test. The diversity of species in aphid assemblages (*H*’) was significantly higher for P3 compared to P4 in 2012 and for P1 compared to P4 in 2014 (Table 4).

**Figure 5:**
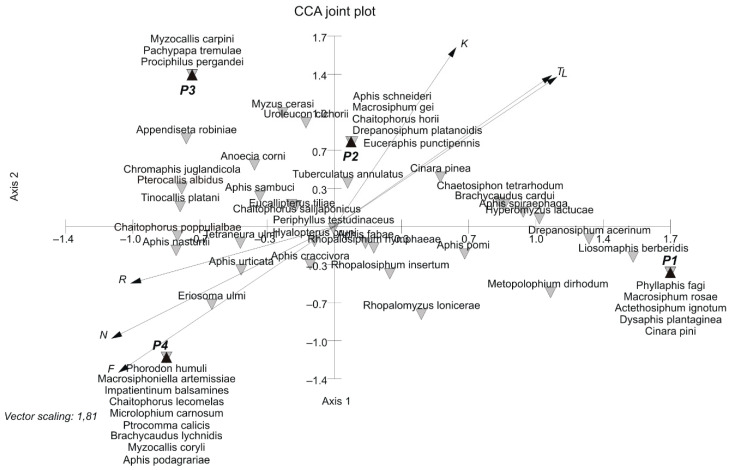
Aphid species co-existence of the four compared aphid assemblages based on CCA ordination and the statistical package MVSP [37] for data analyses with all the recorded aphid species (non-metric multidimensional scaling ordination plot based on Gower’s distance [49] among aphid species and assemblages; to reveal ecological gradients, Ellenberg et al. [35] indicator values were plotted: L—light, T—temperature, K—continentality, F—moisture, R—reaction, N—nitrogen). Legends: Błonie residential district (P1), Park Księżycowy (P2), Park Nad Starym Kanałem on dry soil (P3) and Park Nad Starym Kanałem on moist soil (P4); axis 1—vertical line, axis 2—horizontal line.

**Figure 6:**
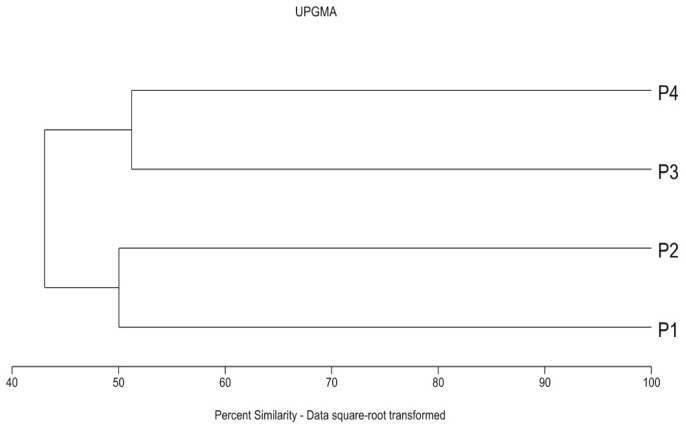
Dendrogram of similarity of the aphid assemblages of the parks (the analysis was performed with the statistical package MVSP [37] and Euclidean distance, UPGMA, as the measure of similarity). Legends: Błonie residential district (P1), Park Księżycowy (P2), Park Nad Starym Kanałem on dry soil (P3) and Park Nad Starym Kanałem on moist soil (P4).

**Figure 7:**
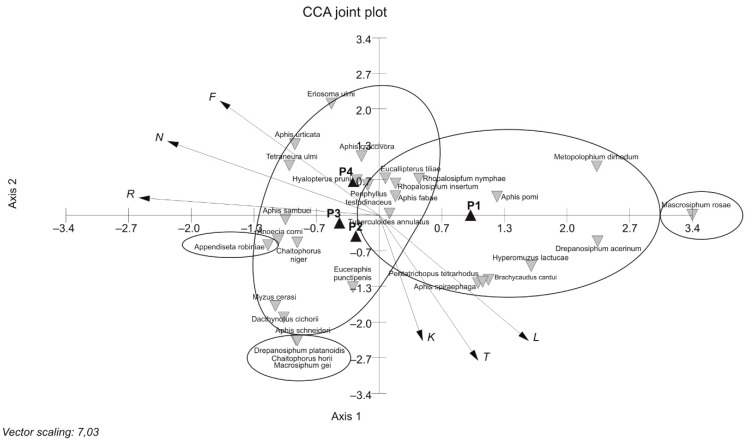
Non-metric multidimensional scaling ordination plot based on Gower’s distance [49] among aphid species and assemblages, and the statistical package MVSP [37] for data analyses (the aphid species in the centre of each ellipse represent a group of so-called co-existence species, taking into account only plant species that are potential aphid hosts, of which at least in one of the parks, the aphids were feeding on one host plant species as a minimum; to reveal ecological gradients, Ellenberg et al. [35] indicator values were plotted: L—light, T—temperature, K—continentality, F—moisture, R—reaction, N—nitrogen). Legends: Błonie residential district (P1), Park Księżycowy (P2), Park Nad Starym Kanałem on dry soil (P3) and Park Nad Starym Kanałem on moist soil (P4); axis 1—vertical line, axis 2—horizontal line.

The diversity of aphid assemblages in the investigated parks was confirmed by numerical analyses. There was no significant correlation between ecological indicators (Table 2, column B), but cluster analysis with Euclidean distance (CCA) and the unweighted pair group method with arithmetic mean (UPGMA) revealed two groups of aphid assemblages: the first included assemblages associated with Park Nad Starym Kanałem (P3, P4), and the second assemblages were found in P1 and P2, surrounded by residential buildings (Figure 5 and Figure 6). Ordination using CCA along axis 1 showed that temperature (T) and light (L) could be the differentiating factors (Figure 5), but statistical evidence supporting this claim based on correlation analysis was only found when aphid assemblages and potential plant host species in the compared parks were taken into consideration (Table 2, columns B and C; Figure 7). Both parts of Park Nad Starym Kanałem form a compact urban green area under the canopy of large trees, creating different light and temperature conditions for aphids colonising plants.

As for Figure 5 and Figure 7, we can observe the effect of environmental variables (long arrows) on the aphid assemblage structure. The direction of each arrow (environmental variable) points to an increase in that particular variable, and the longer the arrow was, the stronger the effect that a particular variable had on the aphid assemblage structure [54]. Aphid species that are located near each other were influenced in a similar manner by the different environmental variables. The ordination is divided into four groups of aphids to assist in generally defining each one of them by the variables within the park and to identify the aphid species in it. Cluster analysis of the distance matrix resulted in a well-separated dendrogram and indicates a separation of aphid species along the first and second axes (Figure 5 and Figure 7). In our study, CCA revealed that the most important factors determining the aphids assemblage composition are temperature (T) and to a lesser degree light (L) and continentality (K), as they were the main contributors to the total variation, as explained by CCA. As for the potential co-existence [50,62] of species in the four compared aphid assemblages, we observed four groups of aphid species separated from each other and from the majority of other species in the different parks, which occurred only in that park. More precisely, in aphid assemblages connected with the parks, we identified four groups of aphids. These were *Mysocallis carpini* Koch, *Pachypappa tremulae* Linnaeus and *Prociphilus pergandei* Smith in P3; *Phorodon humuli* Schrank, *Macrosiphoniella artemisiae* Boyer de Fonscolombe, *Impatientinum balsamines* Kaltenbach, *Chaitophorus leucomelas* Koch, *Microlophium carnosum* Buckton, *Pterocomma salicis* Linnaeus, *Brachycaudus lychnidis* Linnaeus, *Myzocallis coryli* Goeze and *Aphis podagrariae* Schrank in P4; *Aphis schneideri* Börner, *Macrosiphum gei* Koch, *Chaitophorus horii* Takahashi and *Drepanosiphum platanoidis* Schrank in P2; and *Phyllaphis fagi* Linnaeus, *M. rosae*, *Acyrthosiphum ignotum* Mordvilko, *Dysaphis plantaginea* Passerini and *Cinara pini* Linnaeus in P1 (Figure 5). Aphid species positioned between the above-mentioned groups of species in Figure 5 were found either in all parks or at least in two or three of them.

The ordination analysis using CCA (Figure 7) indicated aphid groups other than those in Figure 5, with different habitat preferences, such as *M. rosae*, which was abundant only in one park, P1 (Table 3), and *D. platanoidis*, *C horii* and *M. gei*, also found in one park (P2) with a similar abundance, despite the presence of potential host plants in other parks (Table 3). *Appendiseta robiniae* Gillette was recorded in three parks (P2, P3 and P4) (Figure 7), but its population in the study period was not greater than 20 individuals (the least abundant species), despite the presence of potential host plants in all investigated parks (Table 3).

Factor analysis conducted for aphid assemblages colonising host plants in studied parks indicated that, maybe, spatial isolation due to the existing residential blocks in P1, not found in other parks, was another potential differentiating factor, in addition to temperature and light (Table 2, column C; Figure 7). Tall residential buildings creating a specific ecological barrier may also be responsible for the specific microclimate modifying the behaviour of aphids through increased temperature (*T*), etc. [22,55], and the calculated coefficient of correlation for temperature indicates that this was most likely the only ecological factor significantly differentiating the analysed assemblages of aphids (Table 2, column C), as explained by CCA.

## 4. Conclusions

Our study found that among the trees and shrubs, the most numerous host plants settled by aphids in the studied parks were *Prunus* spp. by the aphid *Hyalopterus pruni*, *Sambucuc nigra* by *Aphis sambuci*, *Acer platanoides* and *A. pseudoplatanus* by *Periphyllus testudinaceus*, and *Philadelphus inodorus* by *Aphis fabae*.

Due to aesthetic reasons, host plants attractive to aphids, but also eagerly colonised by them, such as *Prunus* spp., *Philadelphus inodorus*, *Sambucus nigra* and *Acer* spp., should not be chosen when arranging park plantings in central urban spaces. In addition, *Robinia pseudoacacia*, till now, is not very attractive to aphids in Poland; hence it would seem to be more suitable for taking into consideration in parks than other species.

The most attractive flora/plant communities, taking into consideration the aphid count on leaves of their host plants, were in Park Nad Starym Kanałem on moist soil with vegetation under the canopies of deciduous trees, as well as Park Księżycowy, dominated by lawns and single trees.

Furthermore, factor analysis conducted for aphid assemblages colonising host plants in the studied parks indicated that the most differentiating indicator was spatial isolation due to the existing residential blocks, in addition to temperature and light. Such places do not appear to be an attractive foraging site for aphids.

## Figures and Tables

**Table 1:** Indicators characterising the flora of the examined parks (to reveal ecological gradients, Ellenberg et al. [35] indicator values were plotted: L—light; T—temperature; K—continentality; F—moisture; R—reaction; N—nitrogen).

	Number of Plant Species	Shannon Formula *H*’	Anthropophytisation Rate	L	T	K	F	R	N
P1	161	6.458	52.8%	7.12	5.84	4.31	4.50	6.14	5.13
P2	129	6.025	48.1%	6.84	5.90	4.31	5.00	6.75	6.33
P3	176	6.359	37.1%	7.08	5.80	4.62	4.47	6.22	5.23
P4	163	6.351	19.8%	5.76	5.50	3.76	6.16	6.77	7.15

Legends: Błonie residential district (P1), Park Księżycowy (P2), Park Nad Starym Kanałem on dry soil (P3) and Park Nad Starym Kanałem on moist soil (P4).

**Table 2:** The values of correlation coefficients calculated between ecological indicators proposed by Ellenberg et al. [35] (L—light, T—temperature, K—continentality, F—moisture, R—reaction, N—nitrogen) and the park locations on the ordination axes of CCA, using Pearson‘s test [39] to estimate the significance of differences, taking into account only plant species that are potential aphid hosts, of which at least in one of the parks, the aphids were feeding on one host species in minimum.

Ecological Indicators	(A) All Plant Species		(B) All Aphid Species		(C) All Aphid Species and Potential Host Plant Species	
	No. of Axis		No. of Axis		No. of Axis	
	1	2	1	2	1	2
L	−0.9921 *	0.0622	0.5577	0.7098	0.3791	−0.8423
T	−0.9662 *	−0.2640	0.5745	0.6899	0.3195	−0.9530 *
K	−0.9028	0.3474	0.2059	0.8966	0.0318	−0.8251
F	0.9694 *	−0.1701	−0.5273	−0.7004	−0.3781	0.7829
R	0.6491	−0.4549	−0.5155	−0.2926	−0.5607	0.2581
N	0.8643	−0.3180	−0.5496	−0.5383	−0.4874	0.5680

* *p* ≤ 0.05.

**Table 3:** Mean number of aphids from the given aphid host plants and parks in Bydgoszcz over 2012–2014.

Plant Species	Aphid Species	Parks in Bydgoszcz			
		P1	P2	P3	P4
*Aegopodium podagraria*	*Aphis podagrariae*				34.00
*Acer platanoides*	*Drepanosiphum platanoidis*		30.00		
	*Periphyllus testudinaceus*	468.67	259.01	495.01	1682.99
					
*Acer pseudoplatanus*	*Drepanosiphum acerinum*	403.32	27.00	1.00	
	*Periphyllus testudinaceus*	171.66	536.33	322.33	13.33
					
*Acer negundo*	*Periphyllus testudinaceus*	23.00	39.67		135.00
*Acer saccharinum*	*Periphyllus testudinaceus*	69.66			
*Alnus glutinosa*	*Pterocallis albidus*			103.33	106.01
*Alnus viridis*	*Pterocallis albida*			127.64	
*Angelica archangelica*	*Aphis fabae*				11.00
*Arctium* sp.	*Aphis fabae*		235.33		790.00
*Arctium minus*	*Aphis fabae*				246.34
*Artemisia vulgaris*	*Macrosiphoniella artemisiae*				171.34
*Berberis vulgaris*	*Liosomaphis berberidis*	70.67		1.00	
*Betula pendula*	*Euceraphis punctipennis*	24.00	132.33	91.66	
*Carpinus betulus*	*Myzocallis carpini*			162.68	
*Cerasus avium*	*Myzus cerasi*			972.99	
*Cerasus vulgaris*	*Mysus cerasi*		1645.68		
*Cicorium intybus*	*Uroleucon cichorii*		383.33	33.33	
*Cirsium arvense*	*Aphis fabae*	76.67			83.67
*Cornus alba*	*Anoecia corni*		186.34		
*Cornus sanguinea*	*Anoecia corni*			128.66	48.33
*Corylus avellana*	*Myzocallis coryli*				74.32
*Cotoneaster lucidus*	*Aphis pomi*	174.29	43.66		
*Crataegus monogyna*	*Aphis pomi*	146.67			
*Crataegus sp.*	*Prociphilus pini*			58.67	
*Euonymus europaea*	*Aphis fabae*				119.66
*Fagus sylvatica*	*Phyllaphis fagi*	118.00			
*Galium aparine*	*Myzus cerasi*		63.33		
*Geum urbanum*	*Macrosiphum gei*		40.00		
*Impatiens* sp.	*Impatientinum balsamines*				13.00
*Juglans regia*	*Chromaphis juglandicola*			57.34	25.00
*Lonicera xylosteum*	*Rhopalomyzus lonicerae*	139.67			133.33
*Lapsana communis*	*Uroleucon cichorii*			26.67	
*Malus domestica*	*Aphis pomi*		22.33		84.00
	*Rhopalosiphum insertum*	24.66	22.33	7.00	
					
*Malus sylvestris*	*Aphis pomi*			5.66	
	*Dysaphis plantaginea*	1.67			
					
*Silene latifolia*	*Brachycaudus lychnidis*				34.00
*Philadelphus inodorus*	*Aphis fabae*	689.00	649.34		
*Pinus nigra*	*Cinara pini*	201.00			
*Pinus mugo*	*Cinara piceae*	73.34			
*Pinus sylvestris*	*Cinara pinea*			63.00	
*Populus alba*	*Chaitophorus populialbae*			120.10	193.67
*Populus tremula*	*Pachypappa tremulae*			113.68	
	*Chaitophorus populialbae*			27.33	66.00
					
*Populus nigra*	*Chaitophorus leucomelas*				187.33
*Prunus cerasifera*	*Hyalopterus pruni*	702.09		83.67	329.34
	*Rhopalosiphum nymphaeae*	16.33	188.34	78.01	425.64
					
*Prunus domestica*	*Brachycaudus cardui*	334.67	390.00		
	*Hyalopterus pruni*	139.33	693.33	300.33	153.01
	*Phorodon humuli*				22.67
	*Rhopalosiphum nymphaeae*	470.99			72.66
					
*Phragmites australis*	*Hyalopterus pruni*		2296.33		4016.65
*Rhamnus cathartica*	*Aphis nasturtii*			25.76	69.66
*Ribes aureum*	*Aphis schneideri*		619.00		
*Robinia pseudoacacia*	*Aphis craccivora*	2.67	141.67	4.33	377.00
	*Appendiseta robiniae*		3.67	11.67	1.00
					
*Rosa rugosa*	*Macrosiphum rosae*	1261.00			
	*Metopolophium dirhodum*	64.66			
	*Chaetosiphon tetrarhodum*	180.00	236.66		
					
*Rosa canina*	*Metopolophium dirhodum*				6.33
*Rumex acetosa*	*Aphis fabae*		299.99		
*Rumex crispus*	*Aphis fabae*				
*Rumex hydrolapathum*	*Aphis fabae*			23.33	
*Qercus robur*	*Tuberculatus annulatus*		15.67	83.65	48.67
*Qercus rubra*	*Tuberculatus annulatus*	157.99		274.34	
*Salix fragilis*	*Chaitophorus salijaponicus*				59.00
*Salix ^x^sepulcralis*	*Chaitophorus salijaponicus*		126.33		
*Salix viminalis*	*Chaitophorus horii*		31.67		
	*Chaitophorus salijaponicus*		205.32		
	*Pterocomma salicis*				1344.67
					
*Sambucus nigra*	*Aphis sambuci*		2682.33	499.99	1183.35
*Sonchus asper*	*Aphis craccivora*	37.33			
	*Hyperomyzus lactucae*	129.33			
					
*Sonchus arvensis*	*Aphis craccivora*	10.00			
	*Hyperomyzus lactucae*	60.00			
					
*Sorbus intermedia*	*Aphis pomi*	117.33			
	*Rhopalosiphum insertum*	1.67			41.34
					
*Sonchus oleraceus*	*Hyperomyzus lactucae*		91.67		
*Spiraea* sp.	*Acyrthosiphon ignotum*	11.00			
	*Aphis spiraephaga*	616.34	890.33		
					
*Tilia cordata*	*Eucallipterus tiliae*		0.33	175.01	162.67
*Tilia ^x^Europaea*	*Eucallipterus tiliae*	99.99		314.65	148.02
*Tilia „Euchlora”*	*Eucallipterus tiliae*			197.66	
*Tilia platyphyllos*	*Eucallipterus tiliae*	7.33			
*Tilia tomentosa*	*Eucallipterus tiliae*	58.00		50.33	
*Ulmus glabra*	*Eriosoma ulmi*				379.01
	*Tinocallis platani*			285.66	
					
*Ulmus laevis*	*Tetraneura ulmi*			43.00	897.68
	*Tinocallis platani*				208.00
					
*Ulmus minor*	*Tetraneura ulmi*		524.00		
	*Eriosoma ulmi*		40.00		
					
*Urtica dioica*	*Aphis urticata*		140.00		302.34
	*Microlophium carnosum*				647.67
					
*Viburnum opulus*	*Aphis fabae*		398.67		

Legends: Błonie residential district (P1), Park Księżycowy (P2), Park Nad Starym Kanałem on dry soil (P3) and Park Nad Starym Kanałem on moist soil (P4).

**Table 4:** Aphid assemblage characteristics in the parks of Bydgoszcz in 2012–2014.

	2012						2013				2014				2012–2014			
Parks	P1	P2		P3		P4	P1	P2	P3	P4	P1	P2	P3	P4	P1	P2	P3	P4
Mean number of specimens	3225.34	8220.65		2425.50		6242.00	984.99	2290.32	1246.35	3054.37	3143.67	3820.35	1698.62	5852.33	7354.00	14,331.32	5370.47	15,148.70
Number of species	17	19		18		19	15	15	16	17	21	16	19	24	25	28	28	33
*H*’	3.427	3.192		3.550		3.095	3.0558	3.050	2.960	3.052	3.511	3.350	3.361	3.108	3.873	3.575	3.864	3.648
	P3*-P4										P1*-P4							
Re (%)	P1-P4		P2-P4		P3-P4		No similarities				No similarities				P2-P4			
	56.2		50.9		59.6										51.9			

Legends: Błonie residential district (P1), Park Księżycowy (P2), Park Nad Starym Kanałem on dry soil (P3) and Park Nad Starym Kanałem on moist soil (P4). * Significant differences at *p* ≤ 0.05. *H*’, Shannon–Wiener index; Re, Renkonen‘s coefficient.

**Table 5:** Dominant aphid species over 2012–2014.

	2012				2013				2014				2012–2014			
Aphid Species	P1	P2	P3	P4	P1	P2	P3	P4	P1	P2	P3	P4	P1	P2	P3	P4
*Aphis craccivora*								D3								
*Aphis fabae*					D3	D3				D3		D3				
*Aphis pomi*					D3											
*Aphis sambuci*		D4				D4	D3	D3						D3		
*Aphis spiraephaga*					D4					D3						
*Brachycaudus cardui*					D4	D3										
*Drepanosiphum acerinum*									D3							
*Eucallipterus tilliae*			D3				D4				D3				D3	
*Hyalopterus pruni*	D3	D4	D3	D4		D3		D4		D3		D4	D3	D4		D4
*Macrosiphum rosae*	D3								D4				D3			
*Myzus cerasi*		D3				D3	D4			D3	D4			D3	D3	
*Periphyllus testudinaceus*	D4		D4	D4									D3		D3	D3
*Pterocomma salicis*								D4								
*Tinocallis platani*							D3									
*Tuberculatus annulatus*							D3									

Legends: Błonie residential district (P1), Park Księżycowy (P2), Park Nad Starym Kanałem on dry soil (P3) and Park Nad Starym Kanałem on moist soil (P4). D3, numerous species, subdominant, representing 10.1–20% of the total number of individuals in the given habitat; D4, very numerous species-dominant, accounting for more than 20% of total number of individuals in the given habitat.

## Data Availability

The study did not report any data in a separate link.
